# Photosynthetic Parameters and Oxidative Stress during Acclimation of Crepe-Myrtle (*Lagerstroemia speciosa* (L.) Pers.) in a *meta*-Topolin-Based Micropropagation System and Genetic Fidelity of Regenerated Plants

**DOI:** 10.3390/plants11091163

**Published:** 2022-04-26

**Authors:** Naseem Ahmad, Nigar Fatima, Mohammad Faisal, Abdulrahman A. Alatar, Ranjith Pathirana

**Affiliations:** 1 Plant Biotechnology Laboratory, Department of Botany, Aligarh Muslim University, Aligarh 202002, India; naseembot@gmail.com (N.A.); nigar.fatima77@gmail.com (N.F.); 2 Department of Botany & Microbiology, College of Science, King Saud University, P.O. Box 2455, Riyadh 11451, Saudi Arabia; aalatar@ksu.edu.sa; 3 Plant & Food Research Australia Pty Ltd., Waite Research Precinct, Plant Breeding #46, Waite Road, Urrbrae, SA 5064, Australia; ranjith.pathirana@plantandfood.co.nz

**Keywords:** antioxidant enzymes, carotenoids, conservation, genetic integrity, plant growth regulators, photosynthetic pigments, tissue culture

## Abstract

An improved and stable micropropagation system using the cytokinin, *meta*-Topolin (N6 (3-hydroxybenzylamino purine—*m*T), with nodal explants in *Lagerstroemia speciosa* L. was established. Among the different doses of *m*T, the maximum number of shoots with the highest shoot length was obtained using Murashige and Skoog’s (MS) medium supplemented with 5.0 µM *m*T. The results were consistent throughout the proliferation period, when recorded at week 4, 8, and 12 of being cultured, with an average of 16.4 shoots per nodal explant, and having a mean length of 4.10 cm at week 8. Shoot proliferation rates could be further improved by a combination of 5.0 µM *m*T with 0.5 µM α-naphthalene acetic acid in MS medium; nodal explants produced an average of 24.3 shoots with a mean length of 5.74 cm after 8 weeks of being cultured. Among the five different concentrations of three auxins tested for the rooting of microshoots in MS medium, a 1.0 µM indole-3-butyric acid treatment was the best, with an average of 10.3 roots per microshoot at an average length of 3.56 cm in 93% of microshoots within 4 weeks of being transferred to this medium. A significant reduction of both chlorophyll a and b in leaves during the first week of acclimation corresponded with a high accumulation of malondialdehyde (MDH), indicating that lipid peroxidation affected chlorophyll pigments. From the second week of acclimation, photosynthetic pigment content significantly increased and MDH content decreased. The net photosynthetic rate and leaf carotenoid content showed almost linear increases throughout the acclimation period. Activity of antioxidant enzymes, namely, superoxide dismutase, catalase, and peroxidases, consistently increased throughout the acclimation period, corresponding with the accumulation of photosynthetic pigments, thus demonstrating the role of the improved antioxidant enzymatic defense system during acclimation. A comparison of parent plant DNA with that of the greenhouse acclimated plants using random amplified polymorphic DNA and inter-simple sequence repeat markers showed a monomorphic pattern indicating genetic stability and the suitability of the method for micropropagation of *L. speciosa*.

## 1. Introduction

Queen Crape Myrtle *Lagerstroemia speciosa* Pers. (Lythraceae) is a tropical, medium-large sized tree that can grow up to 25 m and is of commercial importance as an ornamental plant and avenue tree. Tracing its origin to “Tropical Southern Asia” [1], its aesthetic beauty, accompanied by spectacular blooming with large bright pink to lavender flowers with various shades, has made it the “Queen” among flowers where it is grown, and it is also known as “Pride of India”. Having moderately hard timber, the tree is used to make decorative furniture, agricultural implements, pulp, paper, ploughs, boats, fence posts, and so on. The species is also used in biofertilizer manufacturing [2] as a fodder component for afforestation and ecological restoration [3]. The demand for plants is high in many countries because their uses are multipurpose, including plantation establishment, urban horticulture, reforestation, desert rehabilitation, use in furniture, paper, pulp, and timber industries, as well as for medicinal purposes [3,4].

Bioactive compounds in the leaves and fruits of *L. speciosa*, such as ellagitannins (lagerstroemin, reginin A, flosin B, reganin C and D) [5], alanine, methionine, lageracetal, amyl alcohol, and β-sitosterol [6], serve as raw materials in pharmaceutical industries. Additionally, the leaf extracts have anti-oxidant, anti-inflammatory, anti-hypertension, analgesic, diuretic, thrombolytic, anti-cancer [7,8], antifungal, and antiviral properties [9]. The leaves act as a febrifuge and cleansing agent to regulate metabolic processes [10]. Leaf beverages (tea) possess anti-oxidative [11], anti-inflammatory [3,6], anti-hypertensive [8], diuretic, purgative, anti-ulcer, and anti-gout properties [12,13]. Numerous commercial formulations and industrial products (for example, Swanson Banaba extract) are available, including medicinal tablets, capsules, cosmetics, lotions, hair tonics, and hypoglycemic foods. The majority of formulations contain corosolic acid derived from the plant. Corosolic acid is known as “phyto-insulin” or “botanical insulin” due to its insulin-like activities, and its high level in *L. speciosa* implies that this plant may have anti-diabetic therapeutic potential. [14]. Hypoglycemic effects of *L. speciosa* have been proven not only in animal and in vitro studies, but also in clinical studies, for example, using Glucosol^TM^ (standardized to 1% corosolic acid) in Type II diabetes patients [15].

Due to its many valuable characteristics, *L. speciosa* is has been over-exploited in its natural habitat, leading to a scarcity of mature trees [16]. A low density of plants and a scarcity of pollinators means that much of the seed is fertilized through self-pollination, resulting in aborted seed [17]. Viable seeds have a short lifespan (1–2 months), making the establishment of seedlings difficult [18]. Additionally, vegetative propagation of this plant is not widely practiced. To meet these challenges, improved micropropagation methods are required. In vitro propagation of this species is complicated by poor rooting after use of the widely used synthetic cytokinin (CK) and N6-benzylaminopurine (BAP) at the proliferation stage, requiring forced ventilation of vessels [19]. When kinetin (Kn) was used, callus formation was an impediment both at the proliferation and rooting stages, with low numbers of shoots produced [20]. 

*meta*-Topolin (*m*T) (6-(3-hydroxybenzylamino) purine), is an innate aromatic cytokinin (ArCK) originally isolated from *Populus* X *robusta* leaves [21,22]. Being the most biologically active ArCK belonging to the group “Topolins”, *m*T has been found to have distinct advantages in morphogenesis, differentiation, and subsequent rooting and acclimation in the micropropagation industry [21]. It is a hydroxylated form of BAP that produces an *O*-glucoside with relatively higher activity than other derivatives, resulting in less hyperhydricity in tissue culture, thus facilitating easier rooting and acclimation of rooted plantlets [22,23,24].

Recently, the use of *m*T to replace more traditional CKs, such as BAP and zeatin, at the proliferation stage of micropropagation has been reported to result in better rooting and acclimation of plantlets in diverse species, including *Spathiphyllum floribundum* (Araceae) [22], *Ananas comosus* (Bromeliaceae) [25], *Eucalyptus* spp. (Myrtaceae) [26], *Cannabis sativa* (Cannabaceae) [23], *Corylus colurna* (Betulaceae) [27], *Manihot esculenta* (Euphorbiaceae) [28], and *Pterocarpus marsupium* (Fabaceae) [29]. Replacement of BAP with *m*T reduced hyperhydricity, increased the multiplication rate, and resulted in the spontaneous rooting of *Cannabis sativa* [23] and *Eucalyptus* spp. [26]. In comparison with BAP, *m*T supplementation resulted in more shoots with leaves, which had better morphological and anatomical features, and a higher amount of chlorophyll in *Sesamum indicum* (Pedaliaceae) [30], *Oxystelma esculentum* (Asclepiadaceae) [31], and *Vanilla planifolia* (Orchidaceae) [32]. In a study involving 14 contrasting genotypes of gooseberry (*Ribes grossularia*—Grossulariaceae), Kucharska, et al. [33] found that *m*T-supplementation improved the shoot proliferation to 2.8, in comparison with 1.8, which was obtained using the optimal concentration of BAP, which resulted in longer and healthier shoots in subsequent subcultures. Experiments involving multiple CKs, such as Kn and BAP [29], Kn, BAP and thidiazuron [34], and BAP, Kn, and 2-isopentyl adenine [24], have also shown the superiority of *m*T in its capacity to produce healthy shoots that are more amenable to rooting and acclimation. Furthermore, the problem of recalcitrance of kiwifruit genotypes (*Actinidia chinensis—*Actinidiaceae) to the rooting of shoots proliferated in BAP or zeatin media, could be solved by switching to *m*T in culture media for micropropagation [35], and in the management of a large in vitro collection [36].

These advantages of *m*T over some other CKs used in plant tissue culture can be attributed to the lesser oxidative stress experienced by explants cultured on *m*T-supplemented media. For example, plants grown in *m*T-supplemented media had lower H_2_O_2_ content than in BAP-supplemented media in *Daphne mezereum* (Thymelaeaceae) [37]. *Corylus colurna* (Betulaceae) plantlets cultured in a medium containing BAP showed a significantly higher H_2_O_2_ content, enhanced antioxidant enzyme (superoxide dismutase (SOD), ascorbate peroxidase, and catalase (CAT)) activities, and lower chlorophyll than shoots derived from the medium containing *m*T [27].

However, there are no reports on the use of *m*T in *L. speciosa* micropropagation or in any other species in the plant family Lythraceae; therefore, the objective of our work was to optimize the concentration of *m*T in the proliferation and multiplication stages of *L. speciosa* micropropagation, and to investigate if further improvements can be achieved when combined with different auxins. Additionally, we used molecular markers to test if the *L. speciosa* plants regenerated using *m*T in proliferation media were true-to-type, and if the chlorophyll content and oxidative stress status of plants during the acclimation period could improve our understanding of the ability of tissue cultured plants to recover and re-establish themselves.

## 2. Results

### 2.1. In Vitro Establishment and Optimizing Shoot Proliferation and Rooting of Shoots

**Figure 1:**
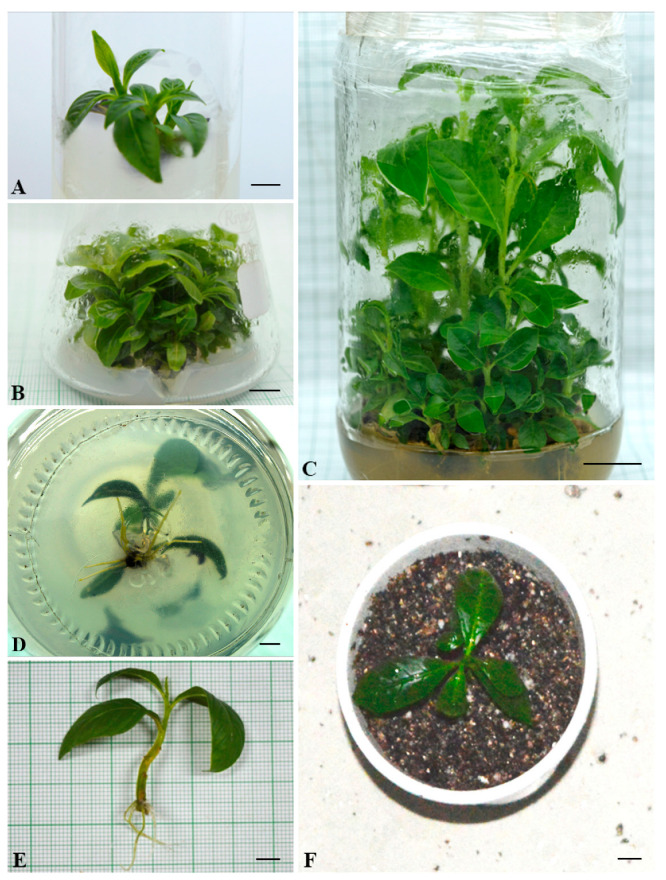
Micropropagation of *Lagerstroemia speciosa* L. (**A**) Multiple shoot induction from nodal segments cultured for 4 weeks using MS medium supplemented with 5.0 µM *m*eta-Topolin (*m*T) (Bar = 0.86 cm); (**B**) mass multiplication of shoots after 8 weeks of being cultured using MS medium supplemented with 5.0 µM *m*T (Bar = 0.89 cm); (**C**) multiplication and elongation of shoots after 12 weeks using the optimal concentration of *m*T (5.0 µM) + NAA (0.5 µM) (Bar = 1.70 cm); (**D**) rooting of in vitro shoots after 4 weeks using MS medium supplemented with 1 µM indole-3-butyric acid (Bar = 0.60 cm); (**E**) rooted microshoot ready for transplantation (Bar 0.71 cm); and (**F**) a 4-week-old acclimatized plant (Bar = 0.71).

Nodal explants collected from mature *L. speciosa* plants were successfully established within 2–3 weeks of initiation. All of the nine *meta*-Topolin (*mT*) concentrations within the range 0.1–15.0 µM in MS medium resulted in the initiation of multiple shoots from the cultured nodal segments (Table 1). There was no shoot production from nodal explants when the medium was not supplemented with *mT*. Supplementation of the medium with 5.0 µM *mT* resulted in a significantly higher number of shoots that were also the longest among the nine concentrations tested (Figure 1A). This result was consistent throughout the period of growth when measurements were taken, from week 4 to 12 (Table 1). With the increase in concentration of *mT* in the medium beyond 5.0 µM, not only did the number of shoots decrease, but also there was an increase in vitrification, resulting in difficulties with rooting and plant acclimation.

When the optimal *m*T concentration for shoot proliferation of *L. speciosa* (5.0 µM *m*T) from the first experiment was combined with different concentrations of three auxins, α-naphthaleneacetic acid (NAA) produced a better proliferation of shoot buds compared with indole-3-butyric acid (IBA) and indole-3-acetic acid (IAA) (Table 2). After 8 weeks in a culture, 0.5 µM NAA in combination with 5.0 µM *m*T resulted in a significantly higher number of shoots than any other combination (Table 2; Figure 1B,C), or with *m*T alone (Table 1), and the highest proportion of explants (95%) producing shoots. Supplementation with IBA or IAA did not have a positive effect on the proliferation of shoot buds, whereas 0.1 and 1.0 µM NAA produced slightly higher numbers of shoots (17.5 and 18.2, respectively) than 5.0 µM *m*T alone (16.4) in 85% of cultures after 8 weeks (Table 1 and Table 2). The shoots produced using MS medium supplemented with 5.0 µM *m*T and 0.5 µM NAA were also significantly longer than all other combinations, except the combination with 5.0 µM *m*T and 1.0 µM NAA (Table 2).

For the rooting experiment, microshoots 3–4 cm in length were harvested from the optimal shoot proliferation medium after 8 weeks of growth and transferred to BM supplemented with one of five concentrations of IAA, NAA, or IBA. The frequency of root initiation varied with auxin concentration (0.5–2.5 µM) (Table 3). BM supplemented with 1 µM IBA produced a significantly higher number of roots per explant and the highest mean length of roots (Table 3; Figure 1D,E). Root development was also the highest in this treatment, with 93% of microshoots producing roots. IBA at 0.5 and 1.5 µM concentrations, and 1.0 µM NAA, also produced high numbers of roots, but the roots in these treatments were much shorter than in the optimum 1 µM NAA concentration. In general, the number of roots produced in IAA-supplemented media was lower than in IBA- and NAA-supplemented media.

### 2.2. Acclimation and Assessment of Photosynthetic Pigments, Antioxidative Enzymes and Genetic Fidelity

**Figure 2:**
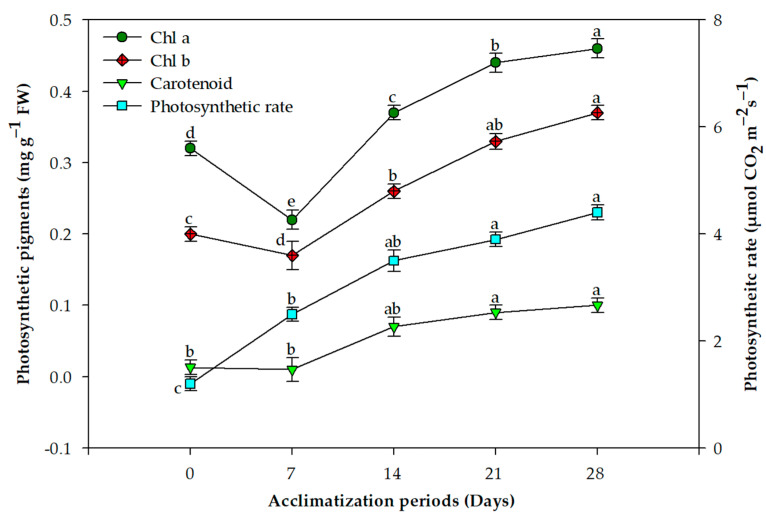
Changes in chlorophyll a, chlorophyll b, carotenoid content, and net photosynthetic rate during the period of acclimation for *Lagerstroemia speciosa* L. plantlets. Line graphs represent mean ± SE of three repeated experiments, each with 20 replications. Means within a category with different letters are significantly different according to Duncan’s multiple range test (*p ≤* 0.05).

**Figure 3:**
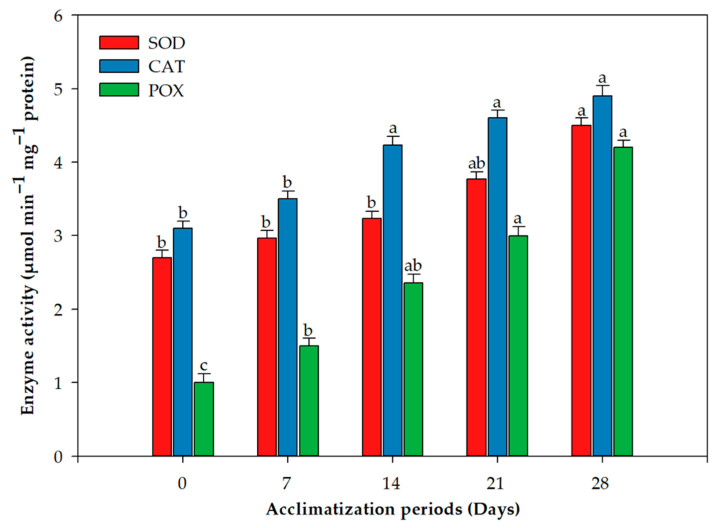
Changes in catalase (CAT), peroxidase (POX), and superoxide dismutase (SOD) activity in leaves of *Lagerstroemia speciosa* L. during the acclimation period. Bars represent mean ± SE of three repeated experiments, each with 20 replications. Means of an enzyme activity with different letters are significantly different according to Duncan’s multiple range test (*p* ≤ 0.05).

**Figure 4:**
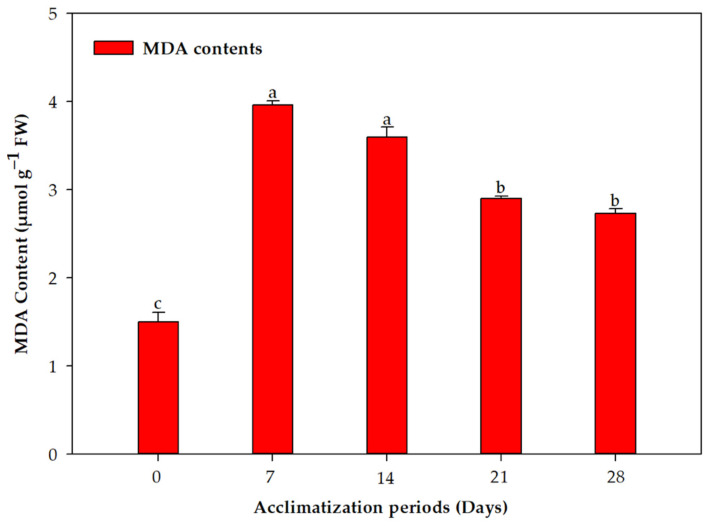
Changes in malondialdehyde (MDA) concentration in *Lagerstroemia speciosa* L. leaves during the acclimation period. Bars represent the mean ± SE of three repeated experiments each with 20 replications. Means in a column with different letters are significantly different according to Duncan’s multiple range test (*p* ≤ 0.05).

Overall, 85% plants survived the acclimation process and transfer to garden soil (Figure 1F). The concentration of chlorophyll a and b behaved similarly over the acclimation period; the concentration declined in the first week and quickly recovered by the third and fourth weeks (Figure 2). This difference was statistically significant for both photosynthetic pigments. On the other hand, the photosynthetic rate and carotenoid concentration displayed a more stable increase without any decline after exflasking (Figure 2). The concentrations of all three assayed antioxidant enzymes increased over the period of acclimation, reaching their maximum in the fourth week, when the plants were transferred to the greenhouse (Figure 3). MDA production, an indication of lipid peroxidation, was low in the rooted plantlets at the time of exflasking, but increased almost threefold in the first week of acclimation, after which it decreased gradually until the plants were transferred to the greenhouse (Figure 4).

**Figure 5:**
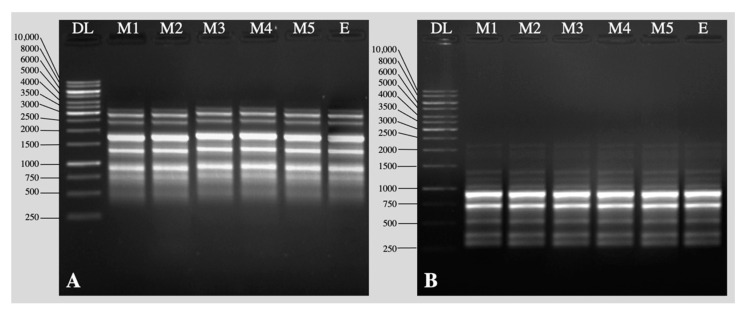
Random amplified polymorphic DNA (RAPD) and inter-simple sequence repeat (ISSR) profiles of *Lagerstroemia speciosa* L. (**A**) Amplified profile from RAPD primer OPA-01; (**B**) amplified profile from ISSR primer UBC-880. DL—DNA ladder; lanes 1–5 randomly selected in vitro plants; lane E—Donor plant.

RAPD and ISSR markers were used for testing the genetic fidelity of micropropagated plants grown in the greenhouse. The mother plant was used as the control. The screening of 18 RAPD primers generated a total of 127 amplified bands with an average of 7.0 bands/primer (Table 4, Figure 5A). Among the 13 ISSR primers screened, 9 primers showed clear, reproducible bands with an average of 7.8 bands/primer (Table 5, Figure 5B). All the bands were monomorphic among the tested plants and the mother plant.

## 3. Discussion

In this study, we report the development of an efficient micropropagation protocol optimized for shoot proliferation from readily available nodal segments of mature trees of *L. speciosa* using *m*T and NAA in a 10:1 proportion, followed by rooting with IBA in MS medium. Previously, when BAP was used at the proliferation stage in *L. speciosa*, rooting was successful when forced ventilation was used in the containers [19]; however, this is costly and impractical for mass-scale operations. In another experiment with this species, the number of shoots per explant obtained with the optimal BAP concentration after 4 weeks was only two [16], compared with the 12 shoots that were obtained using *m*T alone (Table 1) in our research. Furthermore, Lim-Ho and Lee [20] reported that Kn in any concentration, or increasing the BAP concentration beyond 1 mg/L, resulted in callus induction and poor plant regeneration in *L. speciosa*. They managed to increase shoot proliferation using N6-(2-isopentenyl) adenine (2iP) in MS medium; however, CKs with an isoprenoid side chain such as 2iP have the disadvantage of fast oxidation compared with ArCKs [38].

The superiority of *m*T over BAP in micropropagation has been reported recently in other perennial species with medicinal values such as *Oxystelma esculentum* [31], *Pterocarpus marsupium* [29], *Scaevola taccada* [39], and *Bacopa monnieri* [40], as well as in perennial species used in horticulture such as *Daphne mezereum* [37], *Ribes grossularia* [33], *Actinidia chinensis* [35], and *Franklinia alatamaha* [41]. Working on two *Prunus* rootstocks, Gentile, et al. [42] observed that adventitious regeneration from in vitro leaf explants was possible in leaves from shoots growing in media supplemented with *m*T but not BAP; therefore, our research on the micropropagation of *L. speciosa* focused on *m*T. Our first experiment that used *m*T alone in MS media was promising, with a higher number of shoots proliferating than other CKs in *L. speciosa* propagation, based on what has been reported thus far [17,20,21]. When the optimum *m*T concentration of 5 µM was combined with different auxins, we found that the propagation efficiency and quality of shoots could be further improved in combination with 0.5 µM NAA. The synergistic actions of *m*T in combination with auxins in achieving significantly improved shoot multiplication rates have been documented in *Aloe polyphylla* [43], *Uniola paniculata* [44], and *Pterocarpus marsupium* [29].

The problems described in most of the research on micropropagation using BAP, zeatin, and Kn in perennial species, include the low regeneration potential, callus induction at the basal end of the explant, vitrification, and difficulty in rooting micropropagated shoots [36,45,46]. The improvement in shoot proliferation without callus induction when BAP or other CKs are replaced by *m*T has been attributed to its higher activity because of the presence of a hydroxyl group in the N9-position of the purine ring, leading to the formation of the *O*-glucoside conjugate N6(3-O-β-D-glucopyranosyl) benzyladenine-9-glucoside [22,47]. It has also been shown that substitution at the N9 position of CK’s purine ring significantly enhances acropetal transport of the CK, impeding the accumulation of CK glucoside forms in roots [45,46]. This allows for the gradual release of the active form of CK, enhancing the distribution of endogenous CKs in different plant tissues, and thus improving rooting and overall micropropagation efficiency [38]. In our research, as expected, *m*T-induced shoots of *L. speciosa* produced roots with all three auxins tested; however, 1 µM IBA proved to be significantly better in terms of the number of roots, root length, and the percentage of shoots (93%) rooted. This was followed by the successful establishment of a high percentage (95%) of rooted plantlets.

During in vitro culture, plantlets are subjected to diverse stresses that amplify the production of reactive oxygen species (ROS); however, unlike under in vivo conditions, in vitro stress has been studied less in terms of cellular mechanisms to manage stress; namely, constitutive and induced production of radical scavengers, free radical and oxidized-protein enzymatic degradation pathways, and DNA repair mechanisms [48,49]. Oxidative stress in the pathway is associated with the reprogramming of explants to produce fully functional plants results in the peroxidation of lipids, leading to membrane damage, protein degradation, enzyme inactivation, and genetic and epigenetic changes [48,50]. Demonstrating that the elevated amounts of free radicals and their reaction products can lead to morphogenetic recalcitrance in plant tissue culture, Benson [51] emphasized the need for combining studies on oxidative stress with plant development. In our research we focused on the plant acclimation stage, one of the critical stages of micropropagation, particularly because the *m*T -induced proliferation rates were high and produced shoots that were healthy in the optimized media. Furthermore, only a few studies on the oxidative stress in plants during the acclimation period have been conducted.

We combined studies on photosynthetic rates and pigment production with antioxidant enzyme activity during the period of acclimation. Our results revealed that there is a significant decrease in chlorophyll pigments during the first week of acclimation, and this negatively correlated with MDA content in the leaves, indicating high lipid peroxidation at this stage. Activity of all three oxidative enzymes, CAT, POX and SOD, had similar trends throughout the acclimation period; their activity increased as the plants acclimatized. Similar trends in carotenoid accumulation were also observed. Tissue-culture-raised plantlets are exposed to low PPFD. During acclimation, and in the greenhouse, plants are subjected to elevated PPFD that results in photo-inhibition [52]. Carotenoids play a pivotal role in defending the photosynthetic apparatus from photo-oxidative injuries [53], and their elevation along with high activity of ROS scavenging enzymes in *L. speciosa* during acclimation was a result of the plants reacting to the stresses of acclimation, resulting in high survival of our regenerated plantlets. Similar to our results, Goncalves, et al. [54] observed that chlorophyll and carotenoid contents in *Plantago algarbiensis* increased significantly during acclimation compared to the in vitro stage; however, these parameters in *P. almogravensis*, the other *Plantago* species they studied, remained high throughout the in vitro, acclimation, and greenhouse stages [54]. This shows a need to study these processes in the latter stages of micropropagation in different species to understand the outcomes. Similarly, Ahmad, et al. [55] found that the chlorophyll and carotenoid contents of *Decalepis salicifolia* decreased in the first week of acclimation, followed by increases in subsequent weeks; antioxidant enzyme activities also behaved similarly to our results with *L. speciosa*.

The retention of genetic consistency in regenerated plants is critical for any micropropagation system, as well as for conservation programs; therefore, it is important to appraise the genetic consistency of tissue culture-raised plants prior to commercialization or germplasm conservation. In the present experiment, both ISSR and RAPD primers showed high monomorphic patterns, and a way of authenticating the genetic integrity in the micropropagation system was developed. Our results indicate that axillary bud multiplication using *m*T and NAA is a safe method for producing true-to-type plants of *L. speciosa.* The variations observed in the ‘Williams’ banana when topolins or BAP were used, were attributed to excessive subculture cycles [56]. Moreover, the variation was not different between plants regenerated using BAP and *m*T in the ‘Williams’ banana [57] and sesame [30], whereas the regenerants were stable in *Salvia sclarea* [34] and *Daphne mezereum* [37]. There are a number of studies confirming the genetic fidelity of regenerated plants when the cytokinin for axillary bud proliferation is optimized for micropropagation in woody species with medicinal values, such as *Withania somnifera* [58], *Rauvolfia serpentina* [59], and *Cassia alata* [60]. Our results also show that somaclonal variation can be avoided when the growth regulators are used in optimal concentrations and subculturing is performed at regular intervals.

## 4. Materials and Methods

### 4.1. Plant Materials and Surface Sterilization

Juvenile healthy shoots of mature *L. speciosa* plants growing at the Department of Botany of Aligarh Muslim University, Aligarh, Uttar Pradesh, India was collected and washed under running tap water for about 30 min, then treated with the laboratory detergent “Labolene” (Qualigens, Mumbai, India) 5% (*v*/*v*) for 5 min, followed by three to four washes with sterile distilled water. The washed shoots were surface sterilized with HgCl_2_ 0.1% (*w*/*v*) for 4–5 min followed by repeated washing with autoclaved distilled water under aseptic conditions in a laminar air flow cabinet. Nodal explants (1–1.5 cm) were isolated and cultured on sterile initiation media of different compositions as described below.

### 4.2. Basal Media and Culture Environment

The basal growth medium (BM) comprised Murashige and Skoog [61] salts (MS), 3% (*w*/*v*) sucrose, and 0.8% (*w*/*v*) agar (Qualigens Fine Chemicals, Mumbai, India). The medium pH was adjusted to 5.8 prior to adding agar, and the media was then autoclaved at 121 °C for 20 min. The cultures were incubated in a growth room at 25 ± 2 °C with 50 µmol m^−2^ s^−1^ photosynthetic photon flux density (PPFD) provided by white 40 W fluorescent lights (Philips India Ltd., Kolkata, India) at culture container level and 16 h photoperiod.

### 4.3. Shoot Initiation and Proliferation

In the first experiment, nodal explants (axillary buds) were isolated aseptically from surface sterilized shoots and cultured on BM supplemented with different concentrations of *m*T (0.0, 0.1, 0.5, 1.0, 2.5, 5.0, 7.5, 10.0, 12.5, and 15.0 µM) alone. The number of shoots per explant and the length of shoots were recorded on the 4th, 8th, and 12th week. To test if a combination with auxin would further enhance shoot proliferation, the *m*T treatment that gave the best proliferation of shoots (5 µM) was combined with α-naphthaleneacetic acid (NAA), indole-3-butyric acid (IBA), or indole-3-acetic acid (IAA) at five different concentrations (0.1, 0.5, 1.0, 1.5, and 2.0 µM) for the second shoot initiation/proliferation experiment. For this experiment, the shoots were initiated as described above and the number of shoots per explant and the length of shoots were recorded on the 8th week. The shoots from the best treatment in this experiment were used for the rooting experiment described below.

### 4.4. In Vitro Rooting and Plantlet Acclimation

Microshoots (3–4 cm) were dissected aseptically from 8-week-old proliferating axillary bud explants and transferred to root induction media consisting of BM supplemented with one of three auxins viz., IAA, NAA or IBA at 0.5. 1.0, 1.5, 2.0, 2.5 µM concentrations. Root growth was scored after 4 weeks in the culture as rooting percentage, mean number of roots per responding microshoot and mean root length. Microshoots grown on BM with no auxins served as the control.

For acclimation, shoots that had produced roots after 4 weeks of being in the culture under the same conditions as described for shoot organogenesis were removed from the culture tubes, washed to remove agar under tap water, and immediately transferred to polystyrene cups holding sterile Soilrite^TM^ (Keltech Energies Ltd., Bangalore, India). The cups holding plantlets were covered in transparent polythene bags and fed with half-strength MS liquid medium without sucrose at 3-day intervals. The polythene bags were opened gradually over a period of 2 weeks for hardening of the regenerated plantlets. After a further 2 weeks, the acclimatized plants were shifted to pots with regular garden soil and were maintained inside a net house providing 40% filtered sunlight. The number of plants with roots, number of roots per plant, and root length were documented prior to transfer to garden soil.

### 4.5. Photosynthetic Pigments and Net Photosynthetic Rate Assessment

The method described by Mackinney [62] was used to estimate the content of chlorophyll a and b, and the method of Maclachlan and Zalik [63] for carotenoids in leaf tissue. Approximately 100 mg tissue from the interveinal region of leaves in plants at 0 (at the time of exflasking), 7, 14, 21, and 28 (completely developed leaves) days during acclimation were ground in 5 mL acetone (80%) using a mortar and pestle, and the suspension was filtered through Whatman No. 1 filter paper. The supernatant was ground again, filtered, and the entire filtrate was poured into a test tube and the volume adjusted to 10 mL with 80% acetone. For the chlorophyll content, the optical densities (OD) were recorded at 645 and 663 nm using a spectrophotometer (UV-Pharma Spec 1700, Shimadzu, Japan), whereas, for carotenoids, the OD was recorded at 480 and 510 nm.

The net photosynthetic rate in micropropagated plantlets was quantified at 0 (at the time of exflasking), 7, 14, 21, and 28 days after exflasking using an infra-red gas analyzer (IRGA, LICOR 6400, Lincoln, NE, USA). The photosynthetic rate was quantified using the source of net CO_2_ exchange among the leaves with the environment, by enfolding leaves in the leaf compartment of the gas analyzer and recording the rate at which the CO_2_ concentration altered over a 10–20 sec period. The net photosynthetic rate was expressed in µmol CO_2_ m^−2^ s^−1^.

### 4.6. Extraction and Analysis of Antioxidant Enzymes

For the assessment of leaf antioxidant enzyme concentrations, approximately 0.5 g of newly formed leaf tissue were homogenized in a 2.0 mL extraction buffer containing 1% polyvinylpyrrolidone, 1% Triton x-100, and 0.11 g ethylenediaminetetraacetic acid (EDTA) using a pre-chilled mortar and pestle. Filtration of the homogenate via four layers of cheese cloth was followed by centrifugation at 15,000 g for 20 min. The supernatant was used for the enzyme assays. The extraction process was undertaken in the dark at 4 °C.

### 4.7. Assessment of Superoxide Dismutase (SOD) Activity

SOD (superoxide:superoxide oxireductase, EC 1.15.1.1) was quantified according to Dhindsa et al. [64] with minor modifications. SOD activity in the supernatant was assessed by its capability to inhibit photochemical reduction of nitroblue tetrazolium (NBT). The reaction mixture consisted of 0.5 M potassium phosphate buffer, 200 mM methionine, 1 M sodium carbonate, 2.5 mM NBT, 3 mM EDTA, 0.1 mL enzyme extract, 60 µM riboflavin, and 1.0 mL doubled distilled water. The assay mixture in uniform transparent tubes was shaken and placed under a 15 W fluorescent lamp (Phillips, Kolkata, India) for 10 min (25–28 °C). The controls consisted of solutions and enzymes placed in the dark (blank A) and the reaction mixture without enzymes was placed in the light (blank B). Absorbance for samples along with blank B was read at 560 nm against the blank A. A 50% reduction in color was considered as one unit of SOD enzyme activity and expressed in mg protein min^−1^.

### 4.8. Catalase (CAT) Activity Assay

The method described by Aebi [65] with slight modifications was used for the CAT (H_2_O_2_:H_2_O_2_ oxidoreductase: EC 1.11.1.6) activity assay in leaves. The reaction mixture consisted of 0.5 M potassium phosphate buffer, 3 mM EDTA, 0.1 mL enzyme extract, and 3 mM H_2_O_2_ (pH 7.0), and the reaction was allowed to run for 5 min. CAT action was verified via screening H_2_O_2_ loss and quantifying the decline in absorbance at 240 nm. Calculation of CAT activity was carried out by using extinction coefficient (€) 0.036 mM^−1^ cm^−1^ and activity was expressed in enzyme units (EU) mg^−1^ protein min^−1^. One unit of enzyme was defined as the amount of catalase necessary to decompose 1 µmol of H_2_O_2_ per min at pH 7.0 at 25 °C.

### 4.9. Assessment of Peroxidase (POX) Activity

POX (EC 1.11.1.7) content was measured according to Bergmeyer, et al. [66] Fresh leaves (0.2 mg) were collected and homogenized with 5 mL cold phosphate buffer (50 mM, pH 7.8) using a mortar and pestle, and the homogenate was centrifuged at 10,000 g at 4 °C for 20 min. The supernatant collected was maintained at 4 °C until use in the assay. The assay mixture consisted of 0.1 M phosphate buffer (pH 7.8), 4 mM pyrogallol, 3 mM H_2_O_2_, and crude enzyme extract. The assay mixture was transferred to a cuvette and the absorbance was measured at 420 nm using a spectrophotometer (UV-Pharma Spec 1700, Shimadzu, Japan). The enzyme activity was expressed as µmol (H_2_O_2_ consumed) mg^−1^ (protein) s^−1^.

### 4.10. Measurement of Lipid Peroxides

A thiobarbituric acid-reactive-substance assay for measuring malondialdehyde (MDA) was used to assess lipid peroxidation. MDA is a secondary end product of polyunsaturated fatty acid oxidation and is considered to be a useful index of general lipid peroxidation [67]. The method described by Cakmak and Horst [68] was used with modifications. Leaf tissue (0.5 g) was homogenized with 5 mL 0.1% trichloroacetic acid (TCA) and centrifuged at 15,000 g for 5 min. Subsequently, approximately 1 mL supernatant was mixed with 4 mL 0.5% (*w*/*v*) thiobarbituric acid (TBA) in 20% TCA. The mixture was held in boiling water for 30 min, then instantly cooled on ice to end the reaction. The samples were then centrifuged at 12,000 g for 30 min. The absorbance of the supernatant was read at 532 nm using a UV-VIS spectrophotometer (UV-1700 Pharma Spec), and the reading at 600 nm was deducted to compensate for non-specific absorption. The amount of lipid peroxides was measured in terms of MDA level, with an extinction coefficient of 155 mM^−1^ cm^−1^, and expressed as nmol g^−1^ fresh weight.

### 4.11. DNA Extraction and Molecular Marker Techniques

Isolation of genomic DNA from leaf tissues of micropropagated plants, as well as donor plants (mother plant), was conducted using a modified cetyltrimethylammonium bromide (CTAB) technique [69]. Quantification and purity of total DNA was assessed by Nanodrop Spectrophotometer (Implen, Munich, Germany). All the samples were diluted to 25 ng/µL in Milli-Q water and stored at 4 °C until further use. Twenty random amplified polymorphic DNA (RAPD) primers (Operon Kit A Technologies Inc., Ebersberg, Germany) and 13 inter-simple sequence repeat (ISSR) primers (UBC, Vancouver, BC, Canada) were used to examine the clonal fidelity among the regenerated plantlets.

PCR amplification reactions were made in 20 µL volumes containing 1X PCR buffer, 25 mM MgCl_2_ (1.2 µL), 10 mM dNTPs (0.4 µL), 2 µM primers, 3 Unit Taq polymerase (0.2 µL), and 40 ng Template DNA. The amplification of DNA for both RAPD and ISSR analyses was performed in a Thermal Cycler (Biometra, Gottingen, Germany). The PCR amplification program consisted of 45 cycles counting a 94 °C denaturation step of 5 min, an annealing step for 2 min at 35 °C (for RAPD analysis) or 35–58 °C (for ISSR analysis), and a 72 °C elongation of 1 min. A concluding extension was tracked at 72 °C for 10 min. PCR products were analyzed by electrophoresis in 0.8% (*w*/*v*) agarose gels with 4 µL ethidium bromide in a TAE buffer (pH 8.0), run at 60 V for 2 h, and visualized on a UV transilluminator (Bio Rad, Hercules, CA, USA). To regulate the uniformity of the experiments, DNA extraction, PCR reactions and electrophoresis were repeated three times. Distinct and reproducible bands were scored. Bands with the identical movement were considered to be homologous fragments, regardless of intensity.

### 4.12. Statistical Analysis

The two experiments involving *m*T were established with 20 replicates per treatment in a randomized complete block design with six explants per replicate and the experiments were repeated three times. The rooting experiment with three auxins was established with 10 replicates per treatment in a randomized complete block design with a minimum of six microshoots per replicate. Morphological changes among the cultures were observed and documented at regular intervals. The SPSS version 16 (SPSS Inc. Chicago, IL, USA) was employed for statistical analysis of the data. Duncan’s multiple range test (DMRT) at *p* ≤ 0.05 was carried out to examine evidence relating to significant variation among mean values, and the results were expressed as the means ± SE of three experiments.

Research manuscripts reporting large datasets that are deposited in a publicly available database should specify where the data have been deposited and provide the relevant accession numbers. If the accession numbers have not yet been obtained at the time of submission, please state that they will be provided during review. They must be provided prior to publication.

Interventionary studies involving animals or humans, and other studies that require ethical approval, must list the authority that provided approval and the corresponding ethical approval code.

## 5. Conclusions

The present research has successfully established a simple, resourceful, and robust regeneration methodology for micropropagation in *L. speciosa* that will also be useful for ex situ germplasm conservation. The use of the novel ArCK, *m*T, provides a broad platform for in vitro propagation of true-to-type plant material suitable for commercialization, conservation strategies, and pharmaceutical needs. *L. speciosa* shows immense potential for the production of corosolic acid, a “future anti-diabetic drug”, and the plant propagation platform developed in this research could pave the way for commercial production of the plant and its exploitation for bioactives. To the best our knowledge, this *m*T-based in vitro propagation protocol developed in our research is several times more efficient than existing ones.

## Figures and Tables

**Table 1:** Effect of different concentrations of *meta-*Topolin in MS basal medium on in vitro shoot regeneration from nodal explants of *Lagerstroemia speciosa* L.

*meta-*Topolin Concentration (µM)	4 Weeks		8 Weeks		12 Weeks	
	Number of Shoots	Shoot Length (cm)	Number of Shoots	Shoot Length (cm)	Number of Shoots	Shoot Length (cm)
0.0	0.00 ± 0.00 ^j^	0.00 ± 0.00 ^g^	0.00 ± 0.00 ^i^	0.00 ± 0.00 ^f^	0.00 ± 0.00 ^j^	0.00 ± 0.00 ^f^
0.1	2.04 ± 0.04 ^i^	0.88 ± 0.02 ^f^	5.20 ± 0.00 ^h^	0.92 ± 0.16 ^e^	6.85 ± 0.03 ^i^	1.60 ± 0.10 ^e^
0.5	3.20 ± 0.20 ^h^	1.32± 0.08 ^e^	7.62 ± 0.17 ^e^	1.62 ± 0.18 ^d^	10.2 ± 0.18 ^h^	3.36 ± 0.16 ^d^
1.0	4.36 ± 0.36 ^g^	2.50 ± 0.00 ^c^	12.8 ±0.04 ^c^	2.70 ± 0.33 ^bc^	14.1 ± 0.10 ^f^	3.86 ± 0.02 ^e^
2.5	8.10 ± 0.10 ^d^	3.20 ± 0.20 ^b^	14.4 ± 0.18 ^b^	3.08 ± 0.50 ^b^	18.5 ± 0.15 ^c^	4.60 ± 0.00 ^b^
5.0	12.0 ± 0.02 ^a^	3.76 ± 0.06 ^a^	16.4± 0.12 ^a^	4.10 ± 0.10 ^a^	22.4 ± 0.02 ^a^	5.22 ± 0.00 ^a^
7.5	10.0 ± 0.00 ^b^	2.52 ± 0.11 ^c^	11.5 ± 0.15 ^d^	3.12 ± 0.09 ^b^	20.2 ± 0.20 ^b^	4.84 ± 0.08 ^b^
10.0	9.16 ± 0.04 ^c^	2.00 ± 0.00 ^d^	6.50 ± 0.00 ^f^	2.30 ± 0.08 ^cd^	15.6 ± 0.10 ^d^	4.40 ± 0.04 ^b^
12.5	7.10 ± 0.10 ^e^	1.20 ± 0.00 ^e^	5.60 ± 0.17 ^g^	1.50 ± 0.00 ^de^	14.7 ± 0.06 ^e^	3.80 ± 0.40 ^c^
15.0	5.00 ± 0.00 ^f^	0.80 ± 0.00 ^f^	5.00 ± 0.00 ^h^	1.20 ± 0.20 ^de^	11.4 ± 0.04 ^g^	2.96 ± 0.06 ^d^

Values represent mean ± SE of three repeated experiments, each with ten replications. Means in a column with different letters (superscript) are significantly different according to Duncan’s multiple range test (*p* ≤ 0.05).

**Table 2:** Effect of different concentrations of auxins in combination with 5 µM *meta-*Topolin in MS basal medium on shoot initiation and proliferation from nodal segments of *Lagerstroemia speciosa* L. after 8 weeks of being cultured.

Plant Growth Regulators (µM)			% Regeneration	Number of Shoots/Explant	Mean Shoot Length (cm)
IAA	IBA	NAA			
0.1			70	12.5 ± 0.19 ^gh^	4.40 ± 0.40 ^ef^
0.5			75	14.4 ± 0.40 ^e^	5.00 ± 0.00 ^cd^
1.0			60	8.70 ± 0.12 ^j^	4.92 ± 0.02 ^cd^
1.5			70	8.00 ± 0.00 ^k^	4.20 ± 0.00 ^efg^
2.0			65	7.48 ± 0.12 ^l^	3.92 ± 0.02 ^g^
	0.1		80	13.6 ± 0.08 ^f^	4.68 ± 0.08 ^de^
	0.5		85	16.0 ± 0.00 ^d^	5.22 ± 0.02 ^bc^
	1.0		75	14.6 ± 0.16 ^e^	4.34 ± 0.04 ^ef^
	1.5		60	12.9 ± 0.31 ^g^	3.84 ± 0.05 ^g^
	2.0		60	10.0 ± 0.00 ^i^	2.60 ± 0.10 ^h^
		0.1	85	17.5 ± 0.36 ^c^	5.16 ± 0.05 ^bc^
		0.5	95	24.3 ± 0.19 ^a^	5.74 ± 0.06 ^a^
		1.0	85	18.2 ± 0.20 ^b^	5.50 ± 0.00 ^ab^
		1.5	80	15.8 ± 0.30 ^d^	5.20 ± 0.20 ^bc^
		2.0	75	12.0 ± 0.00 ^h^	5.04 ± 0.24 ^cd^

Values represent mean ± SE of three repeated experiments each with ten replications. Means in a column with different letters (superscript) are significantly different according to Duncan’s multiple range test (*p* ≤ 0.05). Plant growth regulators: IAA—Indole-3-acetic acid, IBA—Indole-3-butyric acid and NAA—α-naphthaleneacetic acid.

**Table 3:** In vitro rooting response of *Lagerstroemia speciosa* L. microshoots in basal medium supplemented with indole-3-acetic acid (IAA), indole-3-butyric acid (IBA), and α-naphthaleneacetic acid (NAA) after 4 weeks in the culture.

Plant Growth Regulators (µM)			% Shoots with Roots	Number of Roots Per Microshoot	Root Length (cm)
IAA	IBA	NAA			
0.0			00	0.00 ± 0.00 ^k^	0.00 ± 0.00 ^h^
0.5			85	3.44 ± 0.14 ^g^	2.46 ± 0.06 ^b^
1.0			85	4.36 ± 0.14 ^f^	2.51 ± 0.22 ^b^
1.5			70	3.84 ± 0.04 ^gh^	2.02 ± 0.12 ^d^
2.0			65	3.24 ± 0.06 ^h^	1.98 ± 0.12 ^cd^
2.5			60	2.60 ± 0.10 ^ij^	1.60 ± 0.10 ^ef^
	0.5		65	7.36 ± 0.16 ^c^	2.20 ± 0.20 ^bc^
	1.0		93	10.3 ± 0.12 ^a^	3.56 ± 0.05 ^a^
	1.5		70	8.20 ± 0.20 ^b^	1.76 ± 0.06 ^de^
	2.0		60	6.52 ± 0.21 ^d^	1.54 ± 0.04 ^ef^
	2.5		50	3.20 ± 0.20 ^h^	1.36 ± 0.16 ^f^
		0.5	70	5.69 ± 0.22 ^e^	1.78 ± 0.12 ^de^
		1.0	75	7.24 ± 0.26 ^c^	2.24 ± 0.07 ^bc^
		1.5	65	6.10 ± 0.04 ^de^	2.08 ± 0.20 ^cd^
		2.0	65	2.72 ± 0.13 ^i^	1.54 ± 0.05 ^ef^
		2.5	55	2.20 ± 0.00 ^j^	0.84 ± 0.04 ^g^

Values represent mean ± SE of three repeated experiments, each with 20 replications. Means in a column with different letters (superscript) are significantly different according to Duncan’s multiple range test (*p* ≤ 0.05). Plant growth regulators: IAA—Indole-3-acetic acid, IBA—Indole-3-butyric acid and NAA—α-naphthaleneacetic acid.

**Table 4:** Randomly amplified polymorphic DNA primers (RAPD) used to screen ten micropropagated plantlets of *Lagerstroemia* *speciosa* L.

S. No.	Kit A		
	Primers	Primers Sequences (5′-3′)	No. of Bands
1	OPA 01	GTTTCGCTCG	9
2	OPA 02	TGATCCCTGG	11
3	OPA 03	CATCCCCCTG	8
4	OPA 04	GGACTGGAGT	10
5	OPA 05	TGCGCCCTTC	6
6	OPA 06	TGCTCTGCCC	3
7	OPA 07	GGTGACGCAG	10
8	OPA 08	GTCCACACGG	11
9	OPA 09	TGGGGGACTC	9
10	OPA 10	CTGCTGGGAC	9
11	OPA 11	GTAGACCCGT	8
12	OPA 12	CCTTGACGCA	5
13	OPA 13	TTCCCCCGCT	3
14	OPA 14	TCCGCTCTGG	Nil
15	OPA 15	GGAGGGTGTT	3
16	OPA 16	TTTGCCCGGA	3
17	OPA 17	AGGGAACGAG	9
18	OPA 18	CCACAGCAGT	7
19	OPA 19	ACCCCCGAAG	3
20	OPA 20	GGACCCTTAC	Nil

**Table 5:** Inter-simple sequence repeats (ISSR) primers used to validate the genetic fidelity of micropropagated plantlets of *Lagerstroemia speciosa* L.

S. No.	Primers	Primers Sequences (5′-3′)	Annealing Temperature (°C)	No. of Bands
1	UBC-801	(AT)_8_T	35.0	3
2	UBC-811	(GA)_8_C	49.0	5
3	UBC-825	(AC)_8_T	45.7	Nil
4	UBC-827	(AC)_8_G	49.0	Nil
5	UBC-834	(AG)_8_YT	49.0	9
6	UBC-841	(GA)_8_YC	49.0	Nil
7	UBC-855	(AC)_8_YT	49.0	6
8	UBC-866	(CTC)_6_	55.4	Nil
9	UBC-868	(GAA)_6_	45.7	2
10	UBC-880	(GGGGT)_3_G	49.0	12
11	UBC-889	DBDA(CA)_6_C	45.7	13
12	UBC-891	HVHT (GT)_6_G	45.7	10
13	UBC-900	ACTTCCCCACAGGTTAACAC	58.0	11

## Data Availability

Not applicable.
